# The m6A reader IGF2BP2 promotes esophageal cell carcinoma progression by enhancing EIF4A1 translation

**DOI:** 10.1186/s12935-024-03349-7

**Published:** 2024-05-09

**Authors:** Yuan Li, Zhuya Xiao, Yingying Wang, Daoming Zhang, Zuhua Chen

**Affiliations:** 1 Department of Oncology, Renmin Hospital of Wuhan University, Wuhan University, No. 99 Zhangzhidong Road, Wuhan, Hubei 430060 China; 2 Department of Otolaryngology-Head and Neck Surgery, Renmin Hospital of Wuhan University, Wuhan University, Wuhan, China; 3 Department of Oncology, Tongji Hospital, Tongji Medical College, Huazhong University of Science and Technology, No. 1095 Jiefang Avenue, Wuhan, Hubei 430030 China

**Keywords:** IGF2BP2, ESCC, EIF4A1, m6A, Translation, Oncogene

## Abstract

Esophageal squamous cell carcinoma (ESCC) is one of most prevalent cancers worldwide, especially in China. Lacking in depth mechanism study, effective targets and therapeutics are desperately needed in the clinic. RNA-binding proteins (RBPs) mediate the localization, stability, and translation of the target transcripts and fine-tune the physiological functions of the proteins encoded. Bioinformatics analysis revealed that IGF2BPs were highly expressed in ESCC tissues and at least participated in the regulation of cell proliferation of ESCC cells. Biological researches demonstrated that IGF2BP2 promoted the cell proliferation, migration and invasion of ESCC KYSE30 and KYSE450 cells. IGF2BP2 could bind to EIF4A1 mRNA by recognition of m6A sites and enhanced translation of EIF4A1. IGF2BPs, as m6A reader, IGF2BPs were oncogenic genes in ESCC by regulating the expression of EIF4A1 through m6A sites. IGF2BP2, EIF4A1 and their targets could serve as potential biomarkers and therapeutic targets for ESCC, offering promising novel approaches for the diagnosis and treatment of ESCC.

**Supplementary Information:**

The online version contains supplementary material available at 10.1186/s12935-024-03349-7.

## Introduction

Esophageal squamous cell carcinoma (ESCC) is the most common type of esophageal cancer, especially in developing countries. It represents approximately 90% of the 456,000 new cases of esophageal cancer reported annually [1]. Several high-risk Asian countries, including China, have experienced declining ESCC rates, which may be related to economic development and better nutrition [2]. ESCC is a highly malignant and aggressive tumour that often spreads through the lymphatic and vascular network at an early stage. No targeted therapy has been used in the clinic despite years of research into the molecular characterization of ESCC [3].

Over the past few years, many new molecular factors have been identified that contribute to the development of tumors. These include RNA-binding proteins (RBPs), which play an essential role in modulating post-transcriptional gene expression. It is well known that many RBPs are loosely regulated in various types of cancer [4]. N6-methyladenosine is a common internal modification found in approximately three to five sites in each eukaryote mRNA, and is one of the most common and abundant modifications [4]. m6A modification is modified by the m6A methyltransferases, or writers, such as METTL3/14/16, and, removed by the demethylases, or erasers, including FTO and ALKBH5. It is recognized by m6A-binding proteins YTHDF1/2/3 and IGF2BP1/2/3, also known as “readers” [4].

Emerging evidence implicates m6A RNA modification in the initiation and progression of multiple human cancers. The IGF2BP proteins, which recognise methylated mRNAs and extend their lifespan within stable ribonucleoprotein particles, are emerging as important ‘reader’ proteins in the epi-transcriptomic field. These proteins influence the fate of bound substrates in both normal and pathological conditions, thereby promoting cancer development [5]. For instance, IGF2BP1 binds to PEG10 through m6A modification. Subsequently, IGF2BP1 recruits the polyadenylate binding protein I (PABPC1) to increase the stability of the PEG10 mRNA, resulting in increased PEG10 expression and proliferation of the endometrial cancer cells [6]. In colorectal cancer, IGF2BP3 has been implicated as an m6A reader, and its expression is a predictor of disease progression and poor survival. IGF2BP3 regulates the stability and expression of cyclin D1 and VEGF mRNA by m6A modification [7].

m6A in ESCC is largely unexplored, with only a few studies reported. The m6A demethylase ALKBH5 has been shown to promote the proliferation of ESCC cells, which is linked to a poor prognosis for ESCC patients [8]. The epigenetic modification of the LncRNA LINC00022 by m6A has been found to contribute to tumorigenesis in ESCC. It is a predictor of poor clinical outcome in ESCC patients and is upregulated in primary ESCC samples [9]. . In this study, IGF2BPs, particularly IGF2BP2, were identified as potential regulators in ESCC through systematic screening of RBPs in ESCC samples. The role and underlying mechanisms of IGF2BPs in ESCC were investigated.

## Methods

### Bioinformatics analysis

RNA binding proteins (RBPs) were collected from three independent annotated databases (RBPDB, CISBP-RNA and POSTAR) and a total of 584 RBPs were included in this study, of which 64 RBPs were annotated in all three databases. Two transcriptomic datasets of esophageal squamous carcinoma and normal esophageal tissues were then collected, which were GSE234000 dataset (53 pairs of paired esophageal squamous carcinoma and paraneoplastic normal esophageal tissue) and TCGA vs. GTEx dataset (93 ESCC tissue from TCGA database and 610 normal esophageal tissue from the GTEx database). All data have been reanalyzed according to a uniform pipeline, making the final expression data from the two sources comparable. CRISPR-Cas9 high-throughput screening data were curated from DepMap database, data of IGF2BPs were manually selected from the gRNA libraries targeting 17,670 genes (76,106 gRNAs). Differential expression analysis was performed using Student’s t-test followed by P value adjustment by the “Benjamini-Hochberg” method, utilizing R software (v.4.3.2). Differentially expressed genes (DEGs) were defined as being significantly up or downregulated when P values were < 0.001 and absolute log2 fold-change (LFC) > 1.

### Cell culture

Human ESCC cell lines KYSE30, KYSE70, KYSE150, KYSE180, KYSE410, KYSE450, KYSE510 were cultured in RPMI-1640 (HyClone, USA) medium, supplemented with 10% fetal bovine serum (FBS; GIBCO, USA) and antibiotics (100 U/ml penicillin and 100 mg/ml streptomycin) (Invitrogen, USA). HEK293T cells were cultured in DMEM (GIBCO, USA). All the cells were maintained at 37 °C in 5% CO_2_ cell culture incubator.

### Plasmids

For small hairpin RNA (shRNA) plasmids used in lentivirus-mediated interference, complementary sense and antisense oligonucleotides encoding shRNAs targeting IGF2BP1, IGF2BP2, and IGF2BP3 were synthesized, annealed and cloned into pLKO.1 vector (GENEray, China). Small interfering RNA (siRNA) used in transient transfection were synthesized by GENEray. Plasmids of overexpression of IGF2BP2 and EIF4A1 was constructed into pcDNA3.1 (GENEray, China).

### Cell transfection and lentiviral infection

For transient transfection, cells were transferred with expression vectors using Lipofectamine 3000 (Invitrogen, USA). For lentivirus production, lentiviral vectors were co-transfected into HEK293T cells with packaging plasmid mixture using Lipofectamine 3000 (Invitrogen, USA). Infectious lentivirus particles were harvested at 48 h after transfection, filtered through 0.45 μm PVDF filters and transduced into cells.

### RNA isolation and RT-qPCR

Total RNA was extracted from cells and tissues using Oligotex mRNA mini kit (QIAgen, Germany) according to the manufacturer’s instruction. For RT-qPCR, RNA was reverse transcribed into cDNA by using TransScript II One-Step gDNA removal and cDNA Synthesis SuperMix (Transgen, China). The level of RNA transcripts was analyzed by SYBR Green-based qRT-PCR using a 7900HT fast real time PCR system (Applied Biosystems/Life Technologies, USA). All samples were normalized to GAPDH.

### Western blot and antibodies

Human ESCC cells were washed twice with cold PBS and pelleted. The pellet was resuspended in RIPA buffer (Thermos, USA), and the lysate was obtained by centrifugation at 12,000 g for 10 min. proteins were fractioned by SDS-PAGE, transferred onto PVDF membranes, blocked in 5% nonfat milk or BSA in TBS/Tween-20, and then blotted with specific antibodies. Antibodies used were as follows: anti-IGF2BP1(CST, #8482), anti-IGF2BP2 (CST, #14,672), anti-IGF2BP3 (CST, #57,145), anti-GAPDH (proteintech: 60004-1-Ig), anti-EIF4A1 (Affinity: AF7554), and anti-EIF3B (Affinity, DF8429).

### Cell growth and proliferation assays

Cell viability was detected by adding 10% Cell Counter Kit 8 (CCK-8, DOJINDO, Japan) into the cells plated in 96-well plates and incubated at 37 °C for 2 H at 0, 24, 48, 72 and 96 H. The absorbance of each well was measured by a microplate reader set at 450 and 630 nm. All experiments were performed in triplicates.

For colony formation assay, 500 KYSE30 and KYSE450 cells were maintained in each well of six-well plates and the medium was refreshed every 3 days. Colonies were fixed with methanol after 10 days for KYSE30 and 14 days for KYSE450, then stained with 0.1% crystal violet (Sigma-Aldrich, USA) for 30 min and washed with PBS. The numbers of colonies were counted.

### Transwell migration and invasion assays

Migration assays were performed using a 24-well Transwell chamber system (Corning, USA). 1.5 × 10^5^ cells were seeded in the upper chamber with serum-free culture media in 24-well plates. Media supplemented with 20% FBS were added to the lower chamber. After incubation for 36 H, cells were fixed in methanol for 15 min and then stained with 0.1% crystal violet (Sigma-Aldrich, USA) for 30 min, and counted. Cell invasion assay was performed with BD BioCoat Matrigel Invasion Chambers following the manufacturer’s instructions. Migrated or invaded cells were imaged and counted under a 20× microscope.

### RNA sequencing, RIP sequencing and M6A sequencing

Library preparation and high-throughput sequencing were performed by Epibiotek company (China). RNA sequencing assays were performed in KYSE30 and KYSE450 cells. All samples were sequenced on Illumina NovaSeq 6000 platform. Sequence reads were mapped to the human genome version hg38 by using Illumina sequencing analysis pipeline.

For RIP sequencing, cells were washed twice with PBS, collected and then the pallet was resuspended in IP lysis buffer. The lysate was harvested by centrifugation at 12,000 g for 10 min after incubation for 30 min. antibodies (IGF2BP2, Proteintech:11601-1-AP) and 40ul of protein G beads were added into the lysate followed by incubation overnight at 4 °C. after washed three times with wash buffer, co-precipitated RNAs were extracted by Trizol reagent, ethanol-precipitated with glycogen. The enrichment of RNAs was normalized to IgG.

For m6A sequencing, total RNA was extracted and purified by removing rRNA. After fragmentation, RNA was incubated with m6A antibody for immunoprecipitation according to the standard protocol of the Magna methylated RNA immunoprecipitation m6A Kit (Merck Millipore, Germany). Enrichment of m6A containing mRNA was then analyzed either through high-throughput sequencing or RT-qPCR. For high-throughput sequencing, purified RNA fragments were used for library construction and were sequenced with Illumina NovaSeq 6000 platform. For qRT-PCR, primers to target genes were provided and performed according to the standard protocol of the Magna methylated RNA immunoprecipitation m6A Kit (Merck Millipore, Germany).

### Statistics

Unless otherwise noted, the data are expressed as mean ± SD. The significance of difference was evaluated using Student’s t test or unpaired two-sided t test. The Kaplan-Meier method and the log-rank test were applied to estimate overall survival, progression-free survival, and their differences involved. All of the analysis were performed by Prism. When the P value is < 0.05, the results were considered to be statistically significant.

## Results

### IGF2BP2 and IGF2BP3 were significantly upregulated in ESCC

**Fig. 1:**
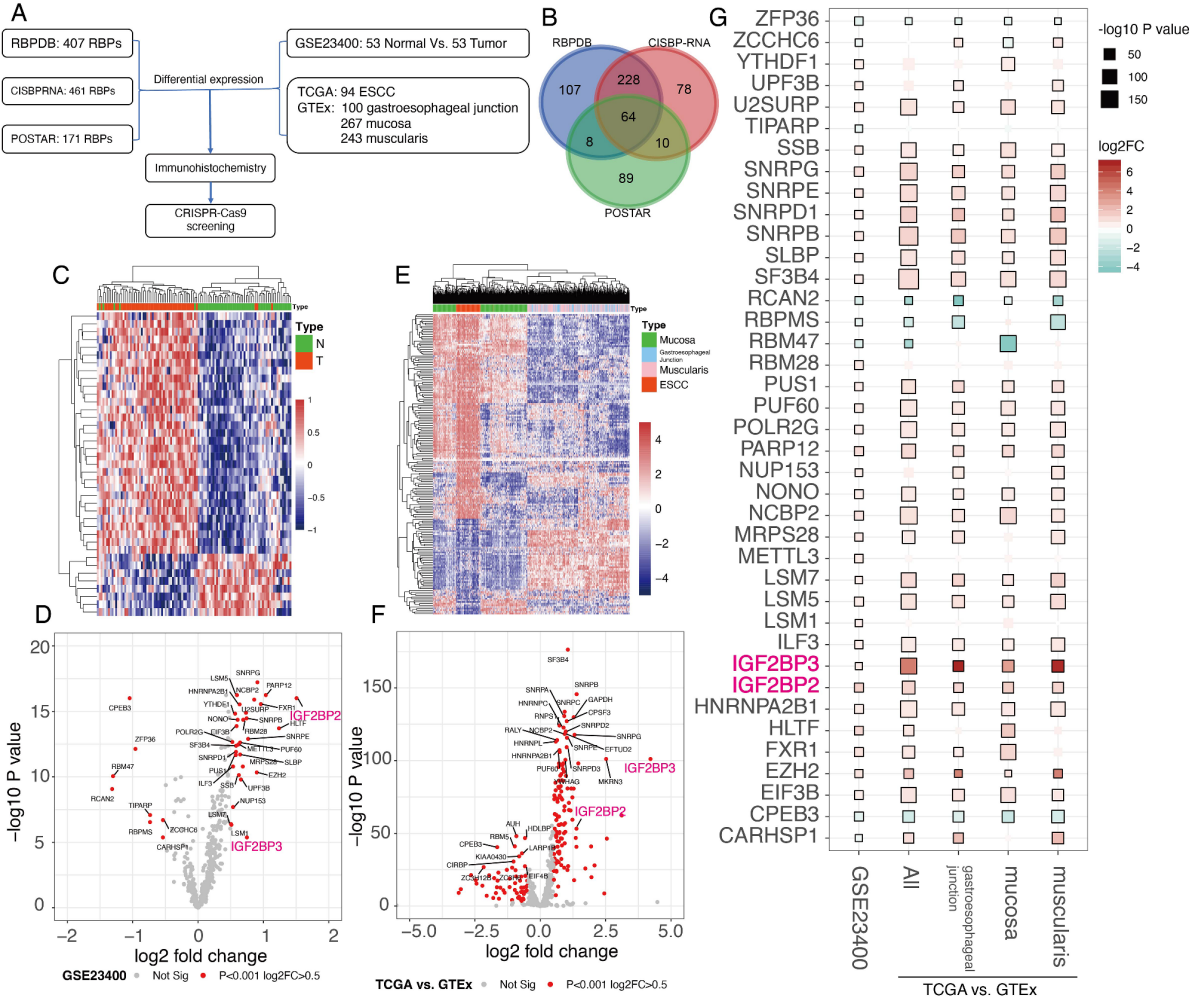
Differentially expressed RBPs in ESCC. (**A**) Analysis pipeline of RPBs in ESCC. (**B**) Venn plot of included RBPs in three independent annotated databases (RBPDB, CISBP-RNA and POSTAR). (**C**) Heatmap of significantly dysregulated RPBs in GSE234000 dataset (53 pairs of paired esophageal squamous carcinoma and paraneoplastic normal esophageal tissue). (**D**) Volcano plot of significantly dysregulated RPBs in GSE234000 dataset. (**E**) Heatmap of significantly dysregulated RPBs in TCGA vs. GTEx dataset (93 ESCC tissue from TCGA database and 610 normal esophageal tissue from the GTEx database). (**F**) Volcano plot of significantly dysregulated RPBs in TCGA vs. GTEx dataset. (**G**) integrated dot plot of significantly dysregulated RPBs in two cohorts. The size of the dot represented significance while the color represented log2 fold change (log2FC)

Epi-transcriptomics is a hot topic of current research, and RNA modification, splicing and editing are inseparable from RNA binding proteins (RBPs). Three independent annotated databases of RBPs (RBPDB, CISBP-RNA and POSTAR) and a total of 584 RBPs were included in this study, of which 64 RBPs were annotated in all three databases (Fig. 1A-B). Two transcriptomic datasets of esophageal squamous carcinoma and normal esophageal tissues were then collected, which were GSE234000 dataset (53 pairs of paired esophageal squamous carcinoma and paraneoplastic normal esophageal tissue) and TCGA vs. GTEx dataset (93 ESCC tissue from TCGA database and 610 normal esophageal tissue from the GTEx database). All data have been reanalyzed according to a uniform process, making the final expression data from the two sources comparable (Fig. 1A). Differential expression analysis showed that significantly differentially expressed RBPs were upregulated in esophageal squamous cancer tissues in both datasets (Fig. 1C and E), and volcano plots showed that IGF2BP2 and IGF2BP3 were significantly upregulated in both datasets (Fig. 1D and F). Finally, integration analysis revealed that IGF2BP2 and IGF2BP3 were two of the most significant genes in both datasets (Fig. 1G), and IGF2BP2 and IGF2BP3 were annotated in all three RBPs databases and are classical RNA binding proteins. In addition, IGF2BP1 of the IGF2BPs family was not significant in differential expression.

Furthermore, immunohistochemistry verified that IGF2BP2 and IGF2BP3 expression was upregulated in ESCC tissues. The expression of IGF2BP2 and IGF2BP3 was significantly higher than that of IGF2BP1 (Fig. S1A-B), and in both datasets, IGF2BP2 and IGF2BP3 were significantly upregulated in ESCC tissues. Validation of these findings were performed using immunohistochemical data from the Human Protein Atlas database with small cohort. IGF2BP1 expression was low in both normal esophageal tissue and tumor tissue, while IGF2BP2 and IGF2BP3 expression was higher in tumor tissue than in normal esophageal tissue (Fig. S1C). This is highly consistent with the results of differential expression analysis, suggesting that IGF2BP2 and IGF2BP3 may have an important role in the development of ESCC, which needs to be further explored in larger cohort.

### IGF2BP2 was a potential prognostic biomarker in ESCC

Survival analysis of IGF2BPs were performed in patients with ESCC in the TCGA database. To exclude the effect of confounding factors such as surgery, a total of 89 ESCC patients with overall survival > 30 days were included. Results showed that the expression of IGF2BPs did not significantly correlate with the survival of ESCC patients (Fig. S2A-C). However, in head and neck squamous carcinoma, which has a similar biological behavior to ESCC, IGF2BP2 was significantly associated with overall survival in head and neck squamous carcinoma patients, with high expression of IGF2BP2 suggesting poorer overall survival (Fig. S2D). In addition, similar results of IGF2BP2 were found in pancreatic cancer and endometrial cancer (Fig. S2E-F.) The lack of significant correlation between IGF2BPs and overall survival in ESCC patients may be related to the small sample size, and it can be speculated from the positive results in other tumors that IGF2BP2 is a potential prognostic marker associated with ESCC.

### IGF2BP2 and IGF2BP3 were involved in regulating the proliferation of ESCC cells

DepMap database offers CRISPR-Cas9 high-throughput screening data to investigate the gene function at genome-wide level in plenty cell lines [10]. In brief, gRNA libraries targeting 17,670 genes (76,106 gRNAs) were used for knockdown of cells in the CRISPR-Cas9 high-throughput screen, and cells were cultured for 21 days after resistance screening, followed by DNA extraction for high-throughput sequencing. When a gene was cell growth-dependent, knockdown of the gene slowed proliferation or increased apoptosis, resulting in a significant downregulation of the gRNA abundance of the gene in the final sequencing results.

**Fig. 2:**
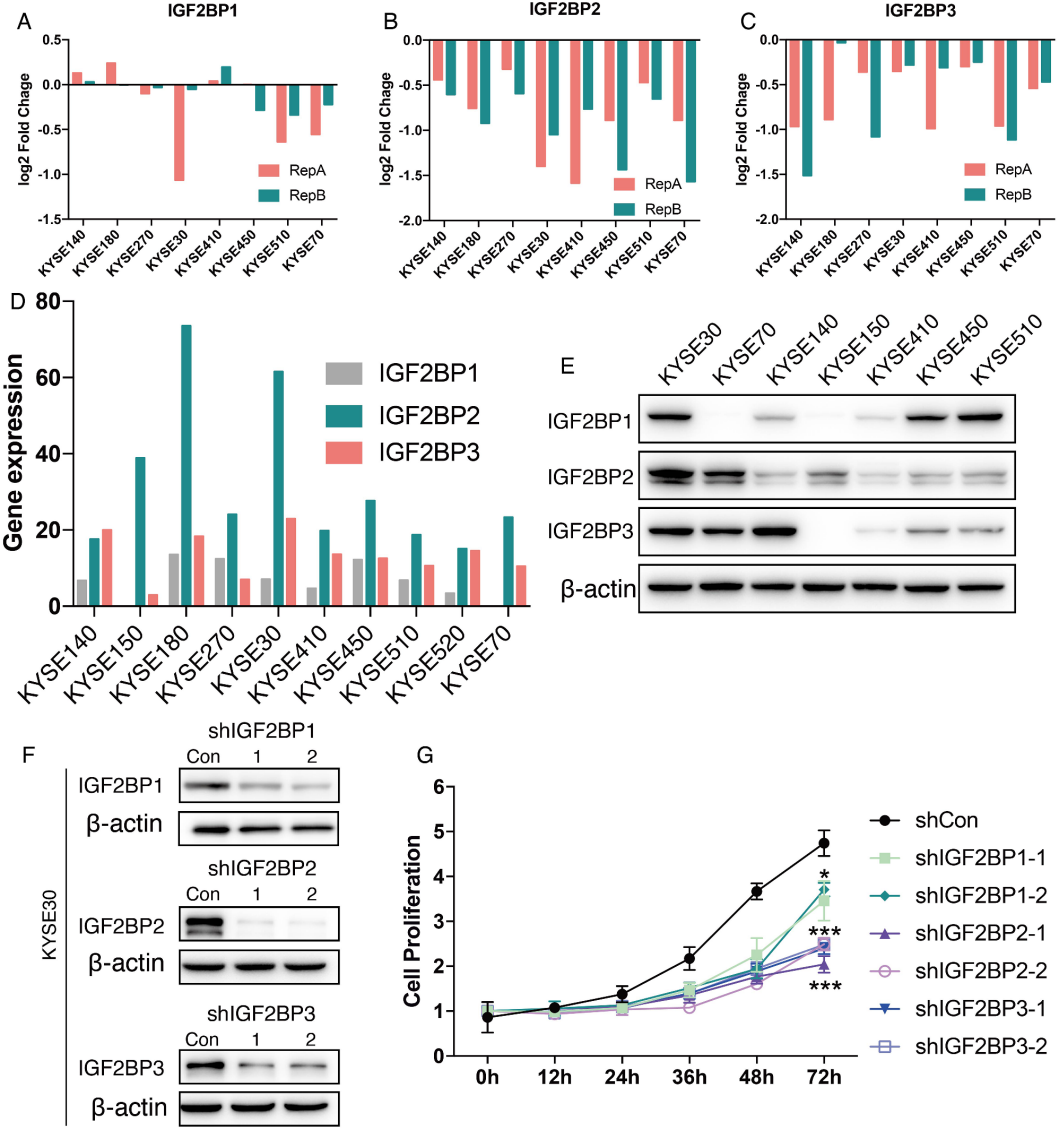
CRISPR-Cas9 high-throughput screening and expression of IGF2BPs in ESCC cells. (**A**-**C**) CRISPR-Cas9 high-throughput screening results of IGF2BP1/2/3 from DepMap database. When a gene was cell growth-dependent, knockdown of the gene slowed proliferation or increased apoptosis, resulting in a significant downregulation of the gRNA abundance of the gene in the fold change. (**D**) RNA expression of IGF2BP1/2/3 in ESCC cell lines. (**E**) Protein expression of IGF2BP1/2/3 in ESCC cell lines. (**F**) Knockdown of IGF2BP1/2/3 in KYSE30. (**G**) Cell proliferation of KYSE30 after knocking down of IGF2BP1/2/3

The results showed that knockdown of IGF2BP2 and IGF2BP3 resulted in significant down-regulation of their gRNA abundance in all eight esophageal squamous carcinoma cells (Fig. 2B-C), while IGF2BP1 was down-regulated in some cells and up-regulated in some cells (Fig. 2A). All these results suggest that IGF2BP2 and IGF2BP3 are growth-dependent genes in esophageal squamous carcinoma cells. Next, the expression of IGF2BPs in ESCC cell lines in the CCLE database were analyzed, and the results showed that the expression of IGF2BP1 was significantly lower than IGF2BP2 and IGF2BP3, and IGF2BP2 was highly expressed in KYSE180 and KYSE30 (Fig. 2D). Western Blot validation in the seven available ESCC cell lines showed consistent results compared to the RNA sequencing data (Fig. 2D-E). IGF2BPs were all highly expressed in KYSE30 cell line (Fig. 2E). Therefore, KYSE30 was selected for cell function experiment by knocking down the expression of IGF2BP1-3, respectively (Fig. 2F), and the results of cell proliferation assay (CCK-8 method) showed that knockdown of IGF2BP1-3 all significantly downregulated the cell proliferation ability of KYSE30, among which, knockdown of IGF2BP2 and IGF2BP3 had a greater effect on proliferation (Fig. 2G). Taken together, IGF2BP2 and IGF2BP3 were significantly highly expressed in ESCC tissues and were involved in regulating cell proliferation in ESCC cell lines.

### Oncogenic role of IGF2BP2 in ESCC cells

**Fig. 3:**
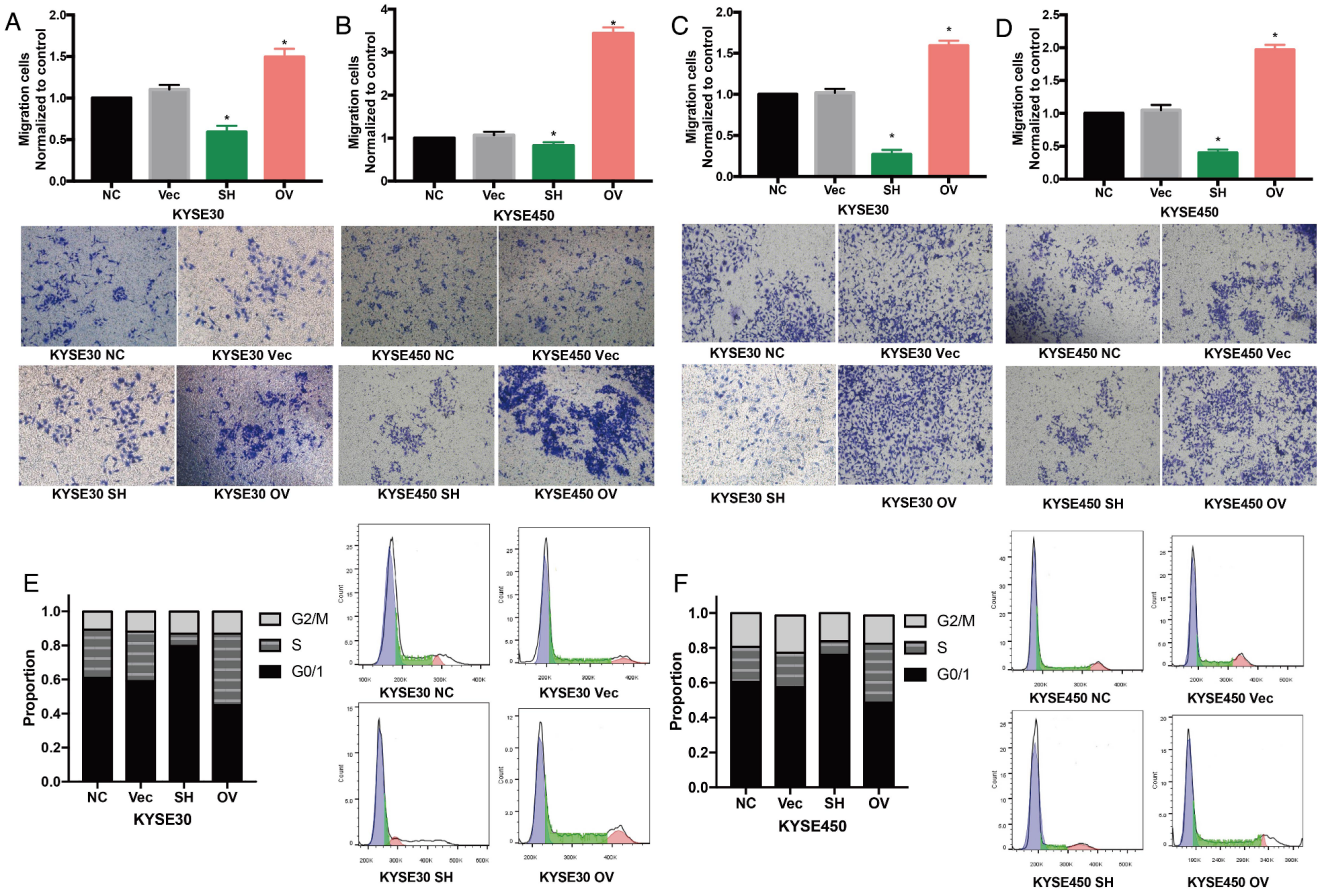
Oncogenic roles of IGF2BP2 in ESCC. (**A**, **B**) Cell migration of KYSE30 and KYSE450 after knocking down and overexpression of IGF2BP2. (**C**, **D**) Cell invasion of KYSE30 and KYSE450 after knocking down and overexpression of IGF2BP2. (**E**, **F**) Cell cycle of KYSE30 and KYSE450 after knocking down and overexpression of IGF2BP2.

Functional experiments by knocking down or over-expression of IGF2BP2 were performed. Results showed that knocking down of IGF2BP2 significantly reduced the number of migrated or invaded KYSE30 and KYSE450 cells (Fig. 3A-D). Accordingly, over-expression of IGF2BP2 significantly increased the number of migrated or invaded KYSE30 and KYSE450 cells (Fig. 3A-D). Cell cycle analysis showed that the proportion of G0/1 phrase KYSE30 and KYSE450 cells significantly increased with lower IGF2BP2 expression and decreased with higher IGF2BP2 expression (Fig. 3E-F).

### IGF2BPs bound RNA by recognizing specific sequences containing m6A methylation in public data

PAR-CLIP (GSE21918) and RIP (GSE90639) sequencing data in the HEK293T cell line targeting IGF2BPs were found in GEO database, while in the HepG2 cell line, m6A methylation sequencing was performed (GES90642). Following the parameters given in the literature, these datasets were reanalyzed and the results showed that 3747, 3211 and 3914 target genes (the target genes identified by both PAR-CLIP and RIP sequencing) for IGF2BP1, IGF2BP2 and IGF2BP3, respectively (Fig. S3A), and a total of 2149 target genes were shared by IGF2BP1/2/3 (Fig. S3A).

Then, the intersection of these 2149 common target genes were matched with the m6A sequencing targets and found that 868 target genes contained m6A methylation sites (Fig. S3B). Meanwhile, the specific sequences (motifs) of IGF2BP1/2/3 binding sites analyzed by HOMER software all contain adenine (A) bases and have high weights (Fig. S3C-E), and the latest literature confirms that IGF2BPs bind RNA molecules precisely by recognizing the specific sequences containing m6A methylation. Finally, Fig. 3F shows the four target genes of the IGF2BPs with high binding peaks in both RIP and m6A sequencing by immunoprecipitation (IP) compared to the Input control. Meanwhile, knockdown of m6A writing gene METTL14 expression was followed by downregulation of the binding peak in m6A sequencing (Fig. S3F). Although these analyses were from different cell lines and not ESCC cell lines, these results demonstrate my ability to integrate and analyze multiple sequencing data on one hand, and demonstrate the close relationship between IGF2BPs and m6A on the other hand, and provide a large number of referenceable targets for this topic.

### Shared potential targets of IGF2BP2 by high-throughput sequencing

**Fig. 4:**
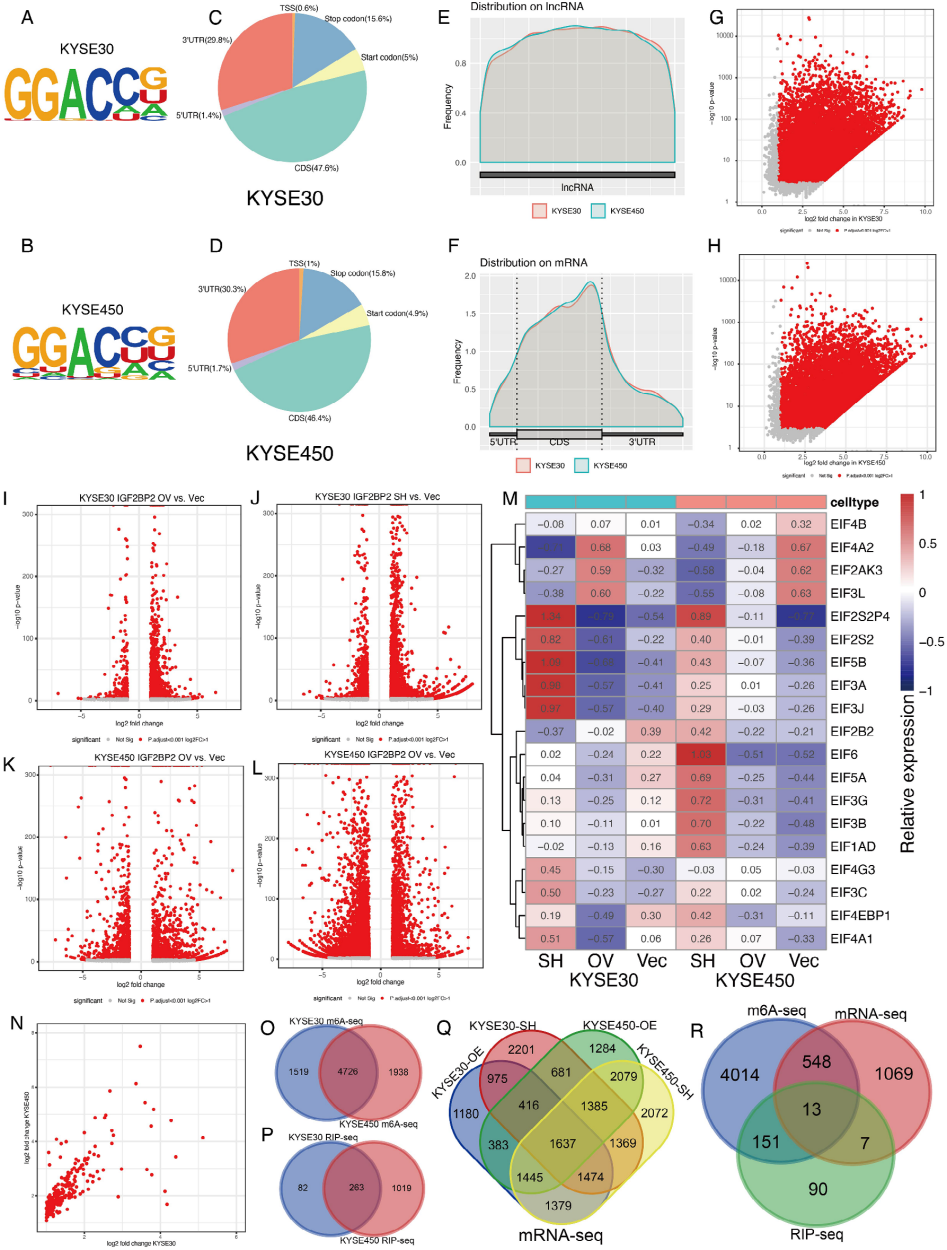
Shared potential targets of IGF2BP2 by high-throughput sequencing. (**A**-**B**) Motif of m6A sites in KYSE30 and KYSE450 by m6A sequencing. (**C**-**D**) Proportions of m6A sites distribution in mRNA in KYSE30 and KYSE450. (**E**-**F**) Distribution of m6A sites in lncRNA and mRNA in KYSE30 and KYSE450. (**G**-**H**) Dot plot of significant m6A sites in KYSE30 and KYSE450. (**I**-**L**) Volcano plots of significantly dysregulated genes after knocking down (SH) and overexpression (OV) of IGF2BP2 by RNA sequencing. (**M**) Heatmap of EIF family genes expression after knocking down (SH) and overexpression (OV) of IGF2BP2. (**N**) Dot plot of enriched IGF2BP binding sites in KYSE30 and KYSE450 by RIP sequencing. (**O**-**Q**) Venn plots of shared targets in m6A sequencing, RIP sequencing and RNA sequencing, respectively (**R**) Venn plot of shared targets in three sequencing data

M6A sequencing analysis were performed in KYSE30 and KYSE450 cells. Motif analysis highlighted that “GGACCG” as the pattern (Fig. 4A-B). Over 45% m6A sites were found in the CDS, followed by nearly 30% in the 3’UTR in both KYSE30 and KYSE450 cells (Fig. 4C-D). Overall, the distribution of the m6a sites found in lncRNAs and mRNAs were largely similar in the two cells (Fig. 4E-F). In detail, 6,245 and 6,664 m6A sites were identified significantly methylated in KYSE30 and KYSE450, respectively (Fig. 4G-H). Lastly, 4,726 m6A genes were shared in both KYSE30 and KYSE450 cells (Fig. 4O).

Transcriptome sequencing was performed in KYSE30 and KYSE450 cells after knockdown and overexpression of IGF2BP2 with vector as control, respectively. Compared with control, 10,138 genes in KYSE30 and 12,840 genes in KYSE450 were significantly differentially expressed after knockdown of IGF2BP2 expression (Fig. 4I-L and Q). Meanwhile, 8,889 genes in KYSE30 and 9,310 genes in KYSE450 were significantly differentially expressed after overexpression of IGF2BP2 expression (Fig. 4I-L and Q). These results were shown in volcano plots. Heatmap demonstrated the EIF family genes which were significantly in at least two groups in KYSE30 and KYSE450 cells (Fig. 4M).

RIP sequencing with antibody of IGF2BP2 were also performed in KYSE30 and KYSE450 cells. A total of 345 genes and 1,282 genes were identified as significant targets in KYSE30 and KYSE450 cells, respectively. Furthermore, 263 genes were shared in the two cell lines (Fig. 4N and P).

M6A sequencing, RNA sequencing and RIP sequencing were performed in KYSE30 and KYSE450 cells. To screening out the most reliable potential targets, which should be significant findings in all tests among the three different sequencing, Venn plots were used to filter the shared genes. However, few gene met this criteria due to limited sample size and statistical efficiency. At last, the concatenation, rather than intersection were used in each type of sequencing data. Then Venn plot with three gene lists, representing potential gene lists from m6A sequencing, RNA sequencing and RIP sequencing, 13 genes were shared in the three sequencing data (Fig. 4R). EIF family genes highlighted as potential targets, which was consistent with previous analysis with public data.

### IGF2BP2 promoted ESCC cell invasion and migration by enhancing EIF4A1 translation

**Fig. 5:**
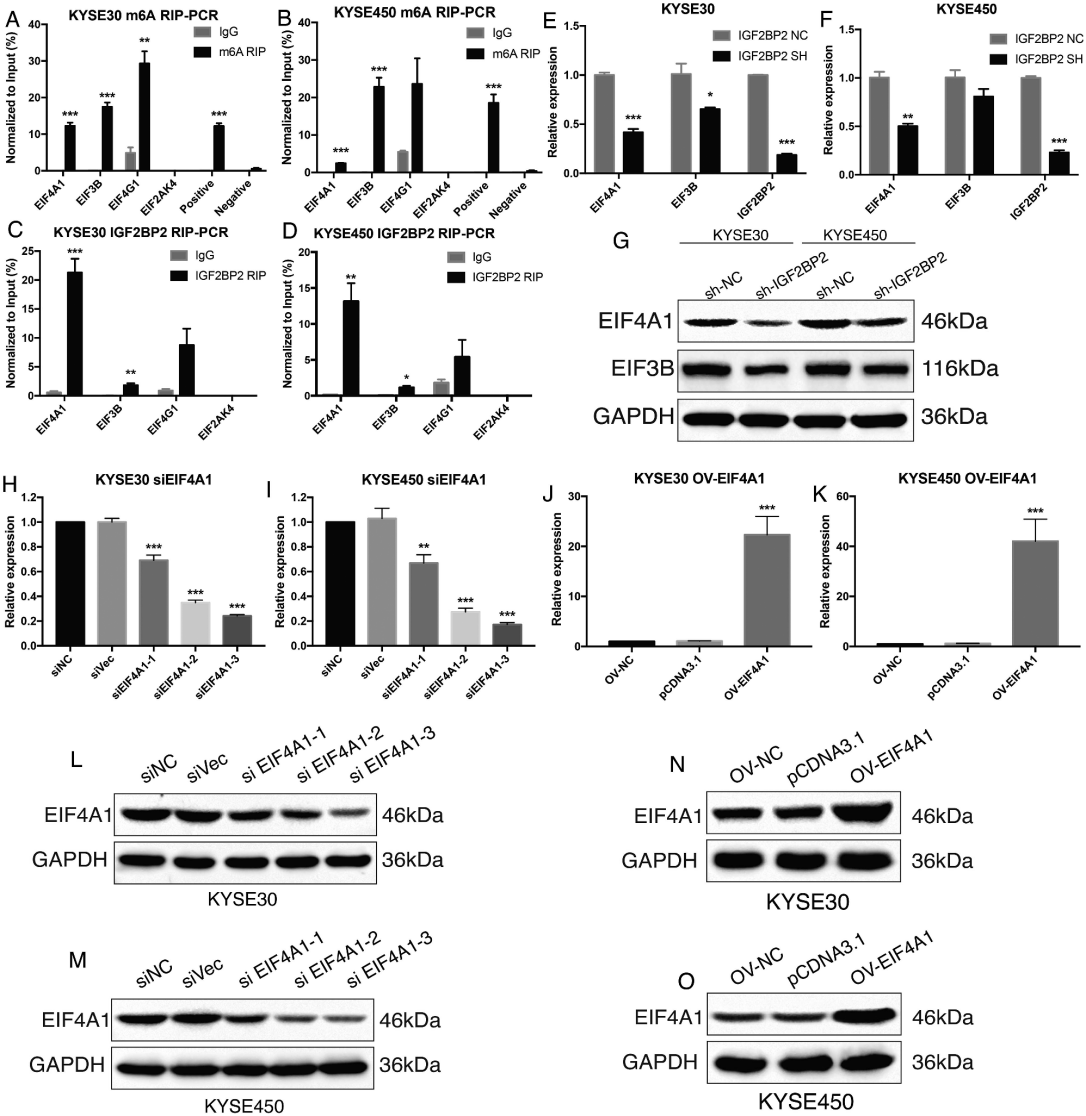
EIF4A1 was screened out as potential target of IGF2BP2 in ESCC cells. (**A**-**B**) m6A RIP-PCR validation of level of m6A in EIF family genes in KYSE30 and KYSE450, respectively. (**C**-**D**) IGF2BP2 RIP-PCR validation of EIF family genes in KYSE30 and KYSE450, respectively. (**E**-**F**) Expression of EIF family genes after knocking down of IGF2BP2 in KYSE30 and KYSE450. (**G**) Protein expression of EIF family genes after knocking down of IGF2BP2 in KYSE30 and KYSE450. (**H**-**O**) Interference of EIF4A1 at mRNA and protein level in KYSE30 and KYSE450, respectively

**Fig. 6:**
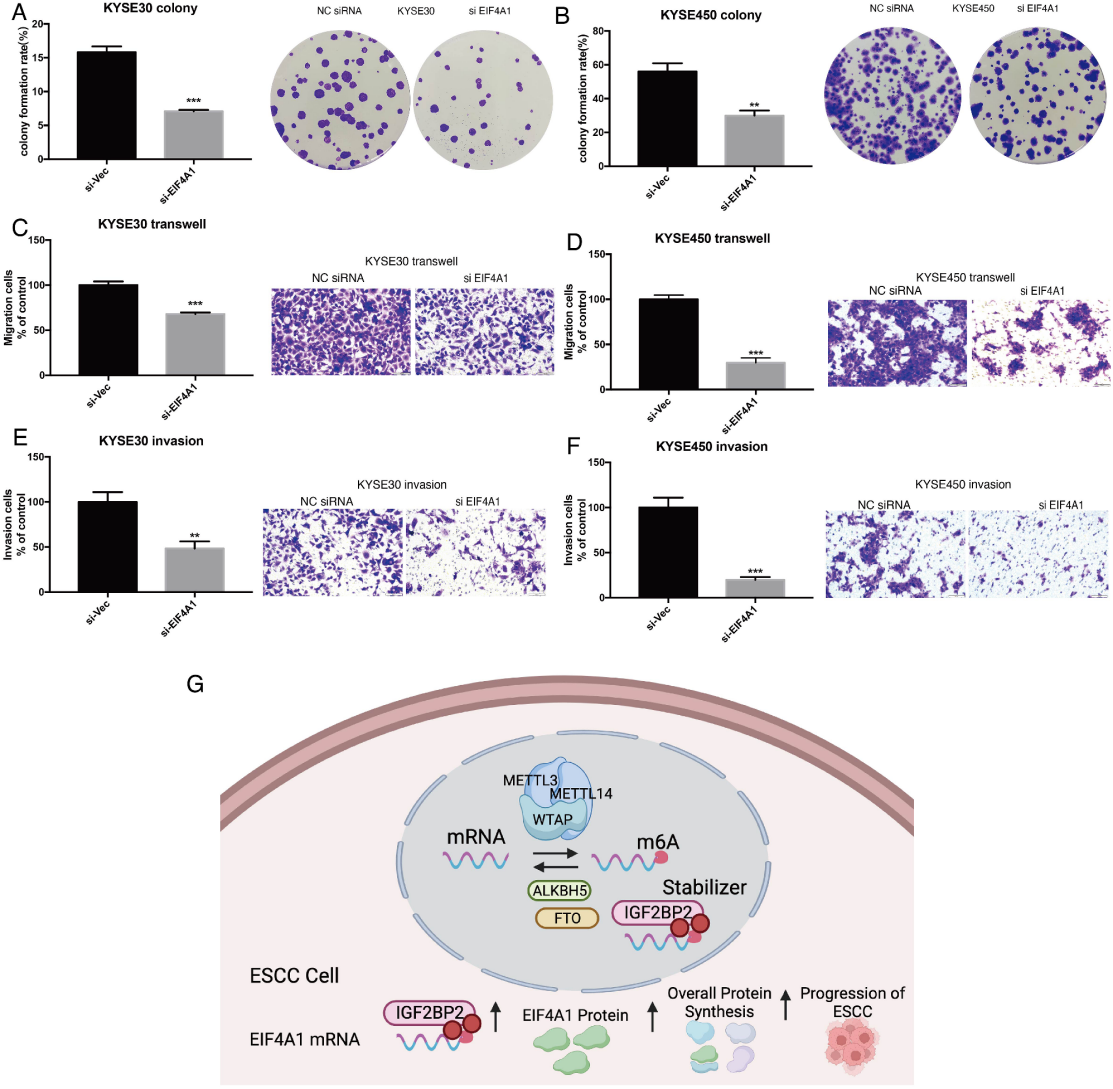
Oncogenic roles of EIF4A1 in ESCC cells. (**A**-**B**) Colony formation after knocking down of EIF4A1 in KYSE30 and KYSE450. (**C**-**D**) Cell migration after knocking down of EIF4A1 in KYSE30 and KYSE450. (**E**-**F**) Cell invasion after knocking down of EIF4A1 in KYSE30 and KYSE450. (**G**) Schematic graph of IGF2BP2 in ESCC cells

To validate the results from high through-put sequencing, m6A RIP-PCR and IGF2BP2 RIP-PCR were performed. In the m6A RIP-PCR, EIF4A1, EIF3B and EGF4G1 were all significantly enriched in both KYSE30 and KYSE450 cells (Fig. 5A-B). However, EIF2AK4 was not significantly enriched (Fig. 5A-B). Next, RIP-PCR targeting IGF2BP2 showed that EIF4A1 and EIF3B were significantly enriched in KYSE30 and KYSE450 cells, whereas EIF4G1 and EIF2AK4 were not statistically significant (Fig. 5C-D). Moreover, when knocking down of IGF2BP2 expression, the expression of EIF4A1 was significantly decreased in both KYSE30 and KYSE450, however, the expression of EIF3B was only significantly in KYSE30 cell (Fig. 5E-F). Similar results were identified at the protein level detected by western blotting (Fig. 5G). Lastly, taken together, EIF4A1 was chose as the target of IGF2BP2 in ESCC cells. Accordingly, interferon of EIF4A1 expression in KYSE30 and KYSE450 cells were successfully carried out for downstream functional experiments (Fig. 5H-O). Results showed that knocking down of EIF4A1 expression significantly attenuated the colony formation, migration and invasion of ESCC cells (Fig. 6A-F). In the rescue experiments, over-expression of EIF4A1, at least partially, reversed the influence of IGF2BP2 knocking down on KYSE30 and KYSE450 cells (Fig. S4A-L). In summary, these results reported the oncogenic roles of IGF2BP2 in ESCC. IGF2BP2 promoted ESCC cell invasion and migration by enhancing EIF4A1 translation, in a m6A methylation dependent manner (Fig. 6G).

## Discussion

Recently, researchers have become interested in the close link between abnormal RBPs and how ESCC develops and progresses [11]. A prognostic model was established by a study which includes seven RBPs - CLK1, DDX39A, EEF2, ELAC1, NKRF, POP7, and SMN1. The analysis showed that the overall survival rate was better in the lower-risk group than in the higher-risk group. These findings provide new insights into the development of ESCC and suggest that these seven RBPs could potentially be used as biomarkers for diagnosing and predicting the prognosis of patients with ESCC.

M6A has been shown to play an important role in human tumorigenesis and is the most common RNA modification in mammalian mRNAs. Yet, its effect on overall protein production in cancer cells is poorly understood. A new mechanism has been discovered involving EIF3C, a subunit of the protein translation initiation factor EIF3, as a direct target of YTHDF1. YTHDF1 enhances EIF3C translation in an m6A-dependent manner by binding to m6A-modified EIF3C mRNA, thereby promoting overall translational output and facilitating ovarian cancer tumorigenesis and metastasis. This novel YTHDF1-EIF3C axis is critical for ovarian cancer progression and could be targeted for the development of cancer therapeutics.

IGF2BP2 belongs to the IGF2 mRNA-binding protein family, which regulates the localization, stability, and translation of mRNA in cells [12, 13]. Research has shown that IGF2BP2 is over-expressed in a number of cancers and is a contributor to tumor progression. While IGF2BP2 is thought to act as a tumor promoter, the mechanisms that regulate its role in RNA metabolism are not well understood [12]. One study [12] found that IGF2BP2 binds to the 3’ untranslated region of the transcript encoding ATP6V1A, a catalytic subunit of the vacuolar ATPase (v-ATPase), and serves as a substrate for the NAD ± dependent deacetylase SIRT1, which regulates how IGF2BP2 affects the stability of the ATP6V1A transcript2. When sufficient levels of SIRT1 are expressed, it catalyzes the deacetylation of IGF2BP2, which can bind to the ATP6V1A transcript but does not mediate its degradation. However, when SIRT1 expression is low, the acetylated form of IGF2BP2 accumulates, and upon binding to the ATP6V1A transcript recruits the XRN2 nuclease, which catalyzes transcript degradation. These findings describe a previously unrecognized role for IGF2BP2 in mediating the degradation of a messenger RNA transcript essential for lysosomal function and highlight how its sirtuin-regulated acetylation state can have significant biological and disease consequences.

Our study included a total of 584 RBPs from three independent annotated databases of RBPs (RBPDB, CISBP-RNA and POSTAR) and IGF2BPs genes were highlighted as significantly differentially expressed RBPs in ESCC. Clinical relevance analysis revealed that IGF2BP2 and IGF2BP3 were significantly upregulated in ESCC and IGF2BP2 was a potential prognostic biomarker in ESCC. In DepMap database, IGF2BP2 and IGF2BP3 are involved in regulating the proliferation of ESCC cells. Functional experiments uncovered the oncogenic role of IGF2BP2 in ESCC cells, promoting the proliferation, migration and invasion of ESCC cells. In both public sequencing data and our sequencing data, IGF2BPs bind RNA by recognizing specific sequences containing m6A methylation, and promote ESCC cell invasion and migration by enhancing EIF4A1 translation.

In summary, IGF2BPs are a family of proteins that were highly expressed in ESCC tissues and played a role in regulating cell proliferation. IGF2BP2, in particular, had been shown to promote cell proliferation, migration, and invasion in ESCC cells. IGF2BP2 functioned as an oncogene at least by binding to EIF4A1 mRNA and enhancing its translation. IGF2BPs and EIF4A1, therefore, are oncogenic genes in ESCC and could serve as potential biomarkers and therapeutic targets for the diagnosis and treatment of ESCC.

### Electronic supplementary material

Below is the link to the electronic supplementary material.

Supplementary Material 1

Supplementary Material 2

## Data Availability

The datasets used and/or analyzed during the current study are available from the corresponding author on reasonable request.
