# Supplementary material for: The m6A reader IGF2BP2 promotes esophageal cell carcinoma progression by enhancing EIF4A1 translation
Source: Cancer Cell Int. 2024 May 9;24:162. doi: 10.1186/s12935-024-03349-7 (PMC11084108; doi:10.1186/s12935-024-03349-7)
Supplement: Supplementary file 1 — Supplementary Material 1 [file 12935_2024_3349_MOESM1_ESM.docx]

Supplementary materials

**Table. siRNA sequence used in this study.**

| Name | Primer | Sequence (5‘-3‘ ) |
| --- | --- | --- |
| NC siRNA | Forward | UUCUCCGAACGUGUCACGUTT |
|  | Reverse | ACGUGACACGUUCGGAGAATT |
| EIF4A1 siRNA-1 | Forward | GCAGAUUGAAUUAGAUCUATT |
|  | Reverse | UAGAUCUAAUUCAAUCUGCTT |
| EIF4A1 siRNA-2 | Forward | AGGUUUCUUUAGUCAUCAATT |
|  | Reverse | UUGAUGACUAAAGAAACCUTT |
| EIF4A1 siRNA-3 | Forward | GGACCAGAUCUAUGACAUATT |
|  | Reverse | UAUGUCAUAGAUCUGGUCCTT |

| **Name** | **Sequence（5’-3’）** | **Size** |
| --- | --- | --- |
| Human EEF1A1 Positive | CGGTCTCAG AACTGT TTGTTTC | 124bp |
|  | AAACCA AAG TGG TCC ACA AA |  |
| Human EEF1A1 negative | GGATGGAAAGTCACCCGTAAG | 95bp |
|  | TTGTCAGTTGGACGAGTTGG |  |
| EIF4A1 | TCATCAACACCCGGAGGAAG | 184bp |
|  | TGCACATCAATGCCTCTGGC |  |
| EIF3B | GACTTTTCTTGGTCTCCTGGTG | 179bp |
|  | ACACACAAGTAGTCTCCGTTCT |  |
| EIF4G1 | GTCAGATCAGTGGAAGCCTCTA | 188bp |
|  | TGCCTTGTAGTCTAGTGGGATC |  |
| EIF2AK4 | AGGTCACTTAACTGGGATGGTT | 191bp |
|  | AAGTGGGATCTCTGAGTTGGTT |  |
| IGF2BP2 | TCTGTCTGGCCTGAGAAGTG  TCTGTCTGGCCTGAGAAGTG | 232bp |
| Homo GAPDH | TCAAGAAGGTGGTGAAGCAGG | 115bp |
|  | TCAAAGGTGGAGGAGTGGGT |  |

**Table. Primers used in this study**
