# Supplementary material for: The m6A reader IGF2BP2 promotes esophageal cell carcinoma progression by enhancing EIF4A1 translation
Source: Cancer Cell Int. 2024 May 9;24:162. doi: 10.1186/s12935-024-03349-7 (PMC11084108; doi:10.1186/s12935-024-03349-7)
Supplement: Supplementary file 2 — Supplementary Material 2 [file 12935_2024_3349_MOESM2_ESM.docx]

**
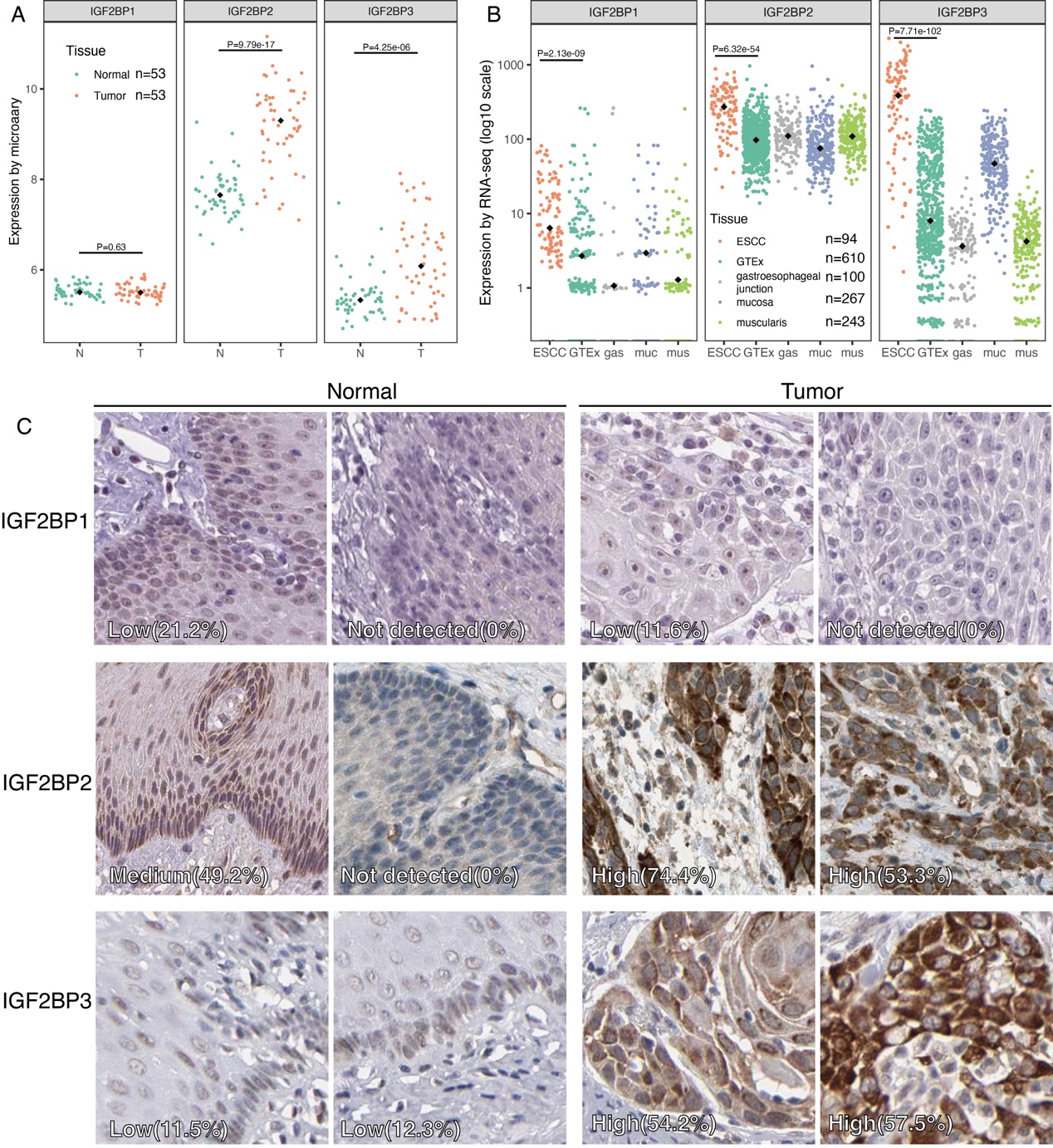
**

**Fig. S1. Expression of IGF2BP1/2/3 in public data.** (A) Expression of IGF2BP1/2/3 in GSE234000 dataset. (B) Expression of IGF2BP1/2/3 in TCGA vs. GTEx dataset. (C) Expression of IGF2BP1/2/3 by immunohistochemistry from the Human Protein Atlas database. Quantitative scores of the positive intensity in the whole slide were provided.

**
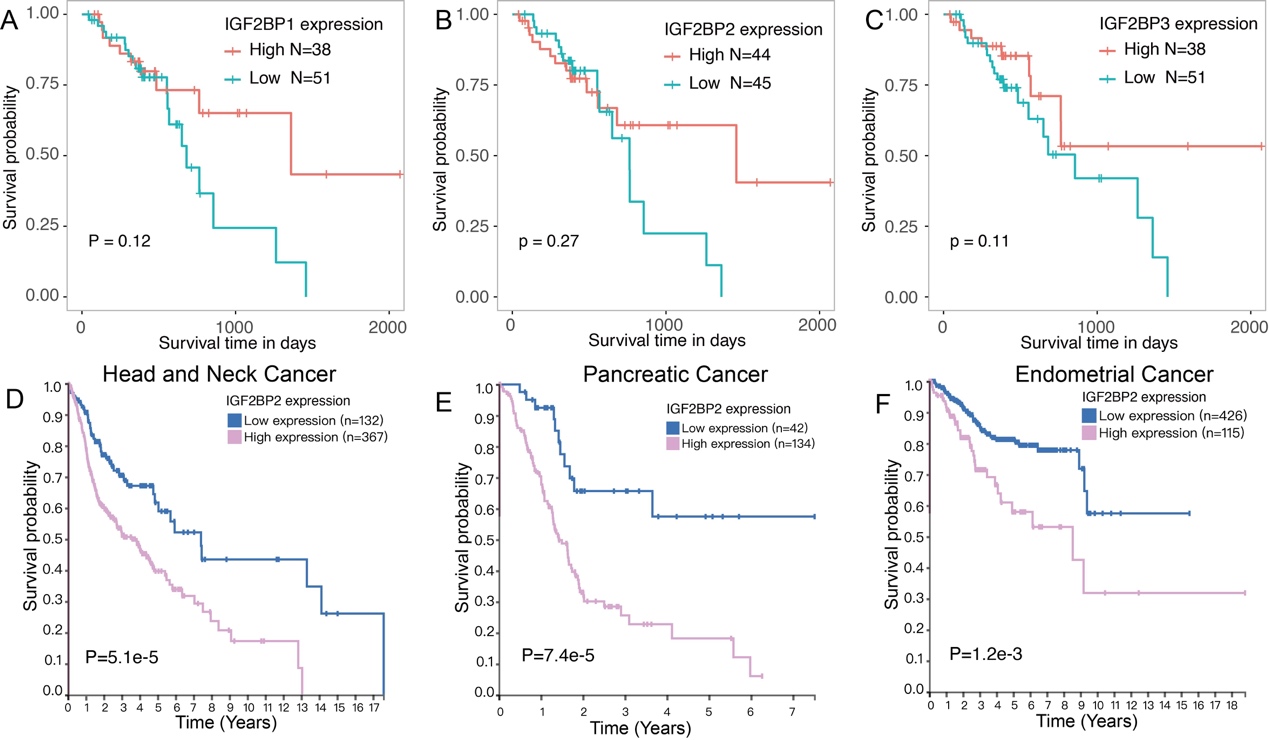
**

**Fig. S2. Prognosis analysis of IGF2BP1/2/3 in cancers from TCGA.** Survival plots of IGF2BP1/2/3 (A-C) in ESCC patients from TCGA. Survival plots of IGF2BP2 in Head and Neck Cancer (D), Pancreatic Cancer (E) and Endometrial Cancer (F).

**
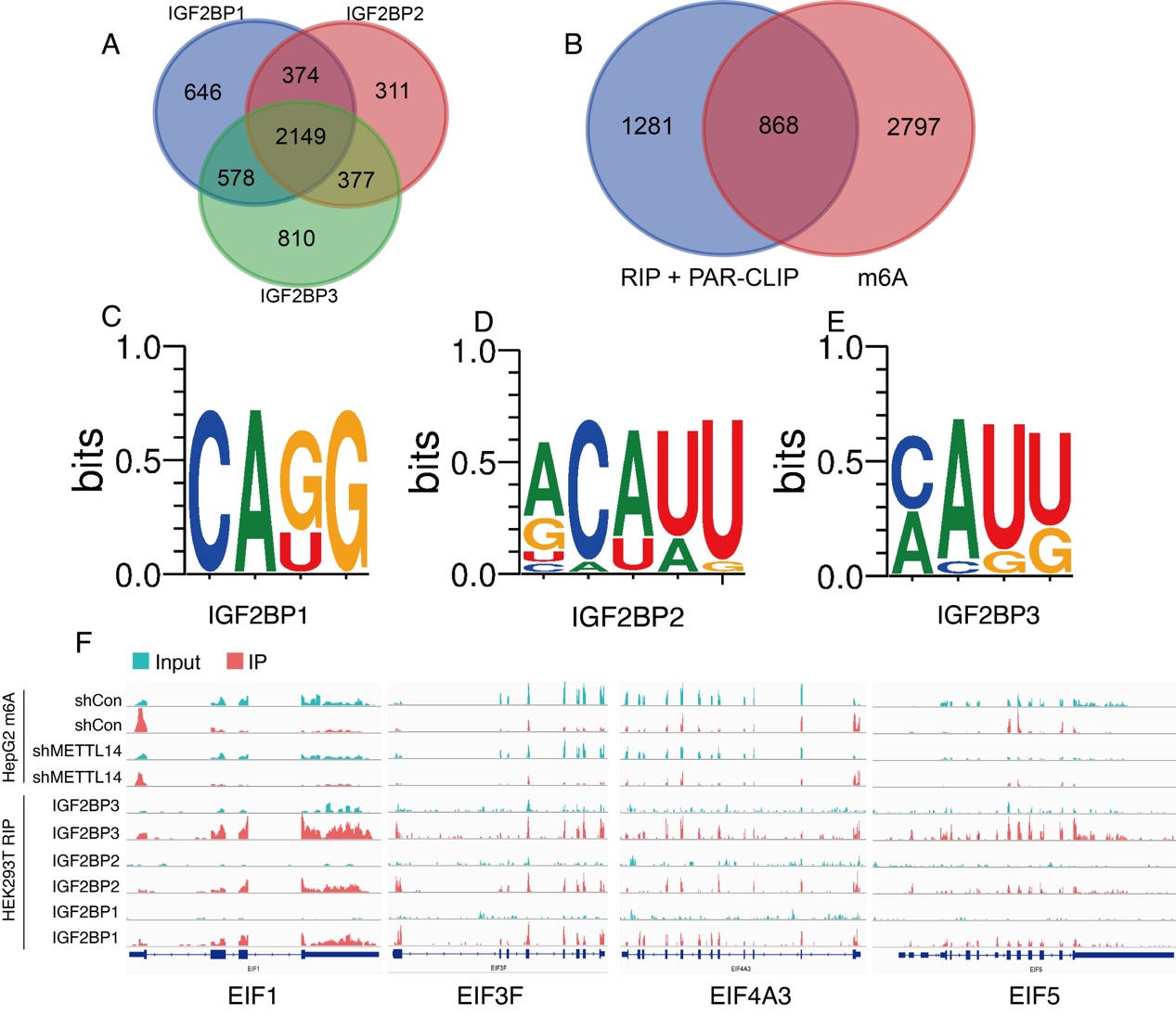
**

**Fig. S3. Potential IGF2BP2 bound targets by m6A motif recognizing in public data.** (A) Venn plot of the target genes identified by both PAR-CLIP and RIP sequencing for IGF2BP1, IGF2BP2 and IGF2BP3. 2149 target genes were shared by IGF2BP1/2/3. (B) Venn plot of the shared target genes (PAR-CLIP and RIP sequencing) and the m6A sequencing targets. (C-E) The specific sequences (motifs) of IGF2BP1/2/3 binding sites analyzed by HOMER software. (F) IGV views of potential targets of IGF2BP1/2/3 from public data.


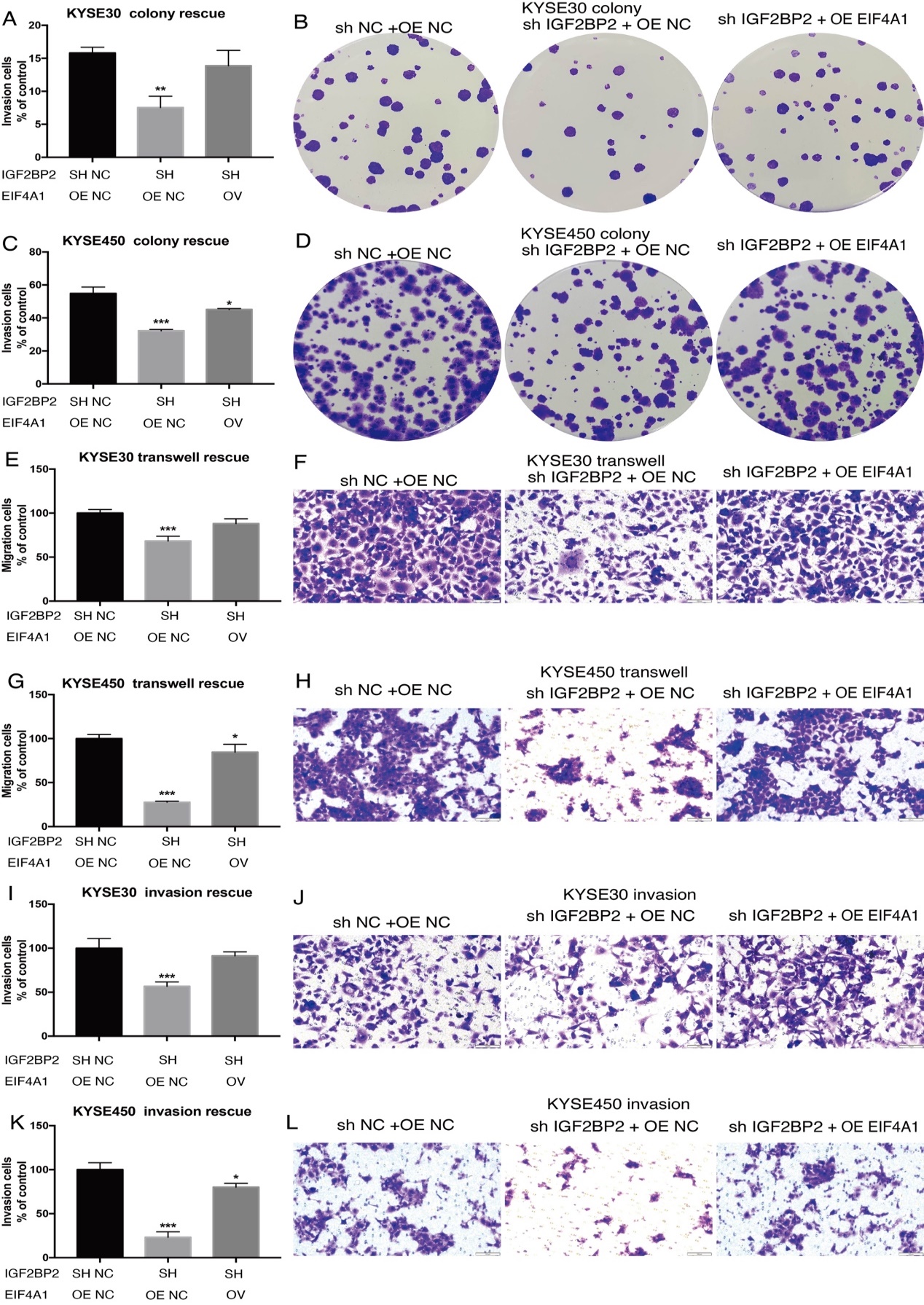


**Fig. S4. Rescue experiments of EIF4A1 and IGF2BP2.** (A-D) Colony formation after interference of EIF4A1 or/and IGF2BP2. (E-H) Cell migration after interference of EIF4A1 or/and IGF2BP2. (I-L) Cell invasion after interference of EIF4A1 or/and IGF2BP2.
